# Late post-endovascular abdominal aortic repair rupture due solely to type II endoleak without other types of endoleak

**DOI:** 10.1093/jscr/rjae792

**Published:** 2024-12-18

**Authors:** Ryo Shimano, Koh Takeuchi, Takuya Komatsu, Junzo Inamura, Suguru Miyazaki, Masafumi Akita

**Affiliations:** Department of Cardiovascular Surgery, Shinmatsudo Central General Hospital, 1-380 Shinmatsudo, Matsudo, Chiba 270-0034, Japan; Department of Cardiovascular Surgery, Shinmatsudo Central General Hospital, 1-380 Shinmatsudo, Matsudo, Chiba 270-0034, Japan; Department of Cardiovascular Surgery, Shinmatsudo Central General Hospital, 1-380 Shinmatsudo, Matsudo, Chiba 270-0034, Japan; Department of Cardiovascular Surgery, Kobari General Hospital, 29-1 Yokouchi, Noda, Chiba 278-0004Japan; Department of Cardiac Surgery, International University of Health and Welfare Mita Hospital, 1-4-3 Mita, Minato, Tokyo 108-8329Japan; Department of Cardiac Surgery, International University of Health and Welfare Mita Hospital, 1-4-3 Mita, Minato, Tokyo 108-8329Japan

**Keywords:** type II endoleak, rupture, AAA, EVAR, warfarin

## Abstract

Rupture of abdominal aortic aneurysm (AAA) due to an isolated type II endoleak (TIIEL) is rarely reported, accounting for less than 1% of all TIIELs; typically, rupture associated with TIIEL is accompanied by type I or type III endoleaks. We report a case of ruptured AAA secondary to TIIEL without any other types of endoleaks, occurring late after endovascular abdominal aortic repair (EVAR). A 77-year-old man with a history of EVAR 11 years earlier presented with abdominal pain. Computed tomography revealed a ruptured AAA, likely due to TIIEL from the lumbar artery. He was on warfarin for atrial fibrillation, and his preoperative PT-INR was 6.05. After administering lyophilized human prothrombin complex concentrate, lumbar artery ligation and aneurysmorrhaphy were performed. Intraoperatively, there was pulsatile bleeding from the lumbar artery, which was sutured closed. No other types of endoleaks were observed. The postoperative course was uneventful, and the patient was discharged home.

## Introduction

Endovascular abdominal aortic repair (EVAR) for abdominal aortic aneurysm (AAA) is widely accepted as a safe and minimally invasive alternative to open repair. However, up to 20% of patients may require further intervention due to complications, such as endoleak, within 5 years after the procedure [1]. Type II endoleak (TIIEL) is the most frequent, and its incidence has been reported as high as 28.9% among the patients treated with EVAR [2]. Since most TIIELs are typically benign and expected to resolve spontaneously, conservative management is recommended unless there is evidence of aneurysm enlargement [3]. Nevertheless, TIIEL can contribute to disease progression, leading to graft migration or sac expansion [4]. Importantly, rupture due to TIIEL is rare, and most reported cases involved concurrent type I or III endoleak. Rupture resulting solely from TIIEL, without other types of endoleak, is infrequently documented, accounting for less than 1% of all TIIEL cases [5]. We present a case of a ruptured AAA attributed solely to TIIEL long after EVAR, which was successfully managed through the lumbar artery ligation and aneurysmorrhaphy.

## Case report

The patient is a 77-year-old man. 10 years ago, he underwent EVAR using an Excluder (Gore) and an endurant left leg (Medtronic), with coiling of the right internal iliac artery for an unruptured AAA measuring 54 × 52 mm and a right common iliac artery aneurysm at another institution. Preoperative contrast-enhanced computed tomography (CT) showed that the inferior mesenteric artery (IMA) had a diameter of 2.86 mm, and six pairs of lumbar arteries were observed, with the largest having a diameter of 2.08 mm. No intervention was performed on these arteries.

5 years later, additional treatment with an Excluder leg extension and coiling of the left internal iliac artery was performed to address type Ib endoleak originating from the left side and the expansion of the AAA to 81 × 82 mm. Subsequently, gradual aneurysm enlargement was observed during the follow-up period; however, because the seals and overlaps of the stent grafts were adequate, he was managed with observation.

**Figure 1:**
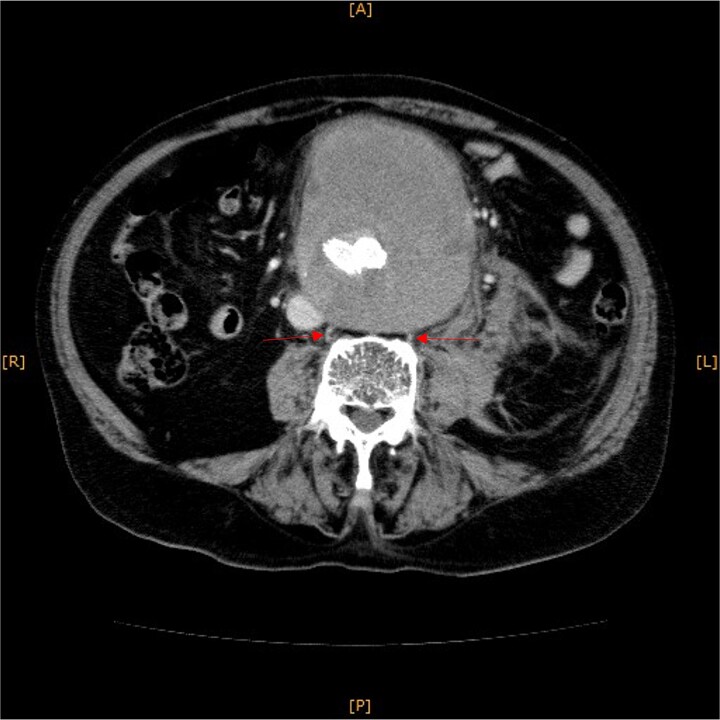
Contrast-enhanced CT revealed an AAA measuring 118 × 107 mm and left retroperitoneal hematoma. One pair of lumbar arteries was enhanced only in the delayed phase (red arrows). Intraoperatively, pulsatile bleeding was observed from the left one of this pair.

**Figure 2:**
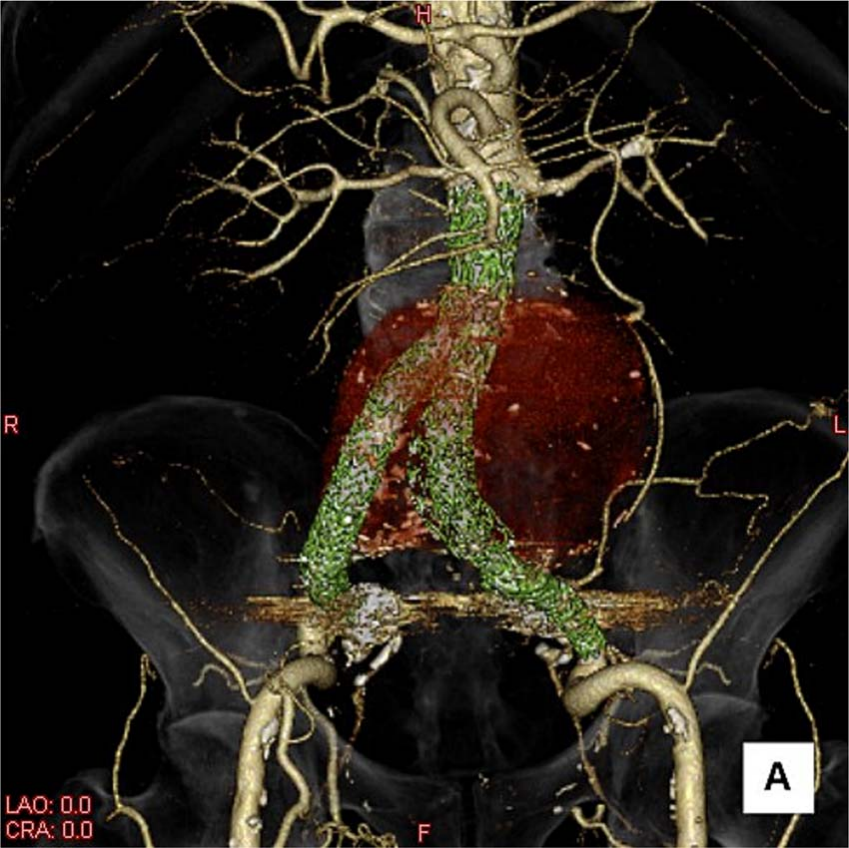
A three-dimensional image. The AAA is shown in transparent red, and the framework of the stent grafts is displayed in green. The landing zones and overlaps of the stent grafts were satisfactory.

**Figure 3:**
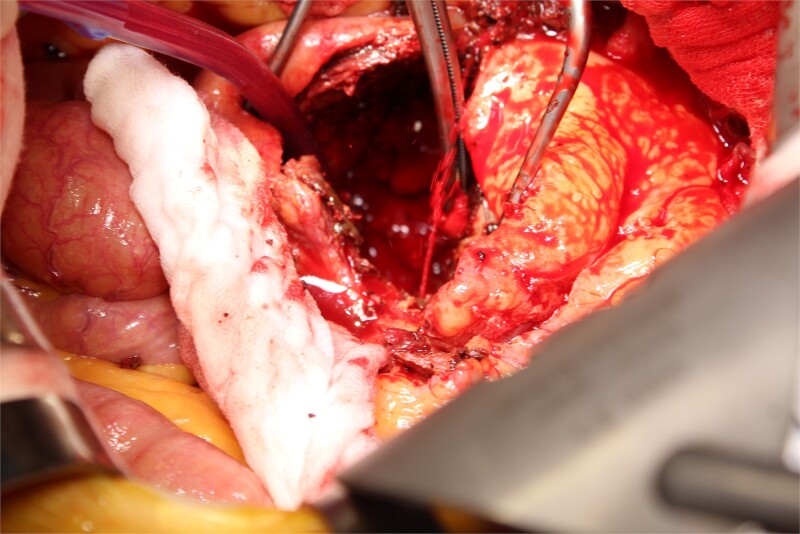
A photo taken from the cranial side of the patient. A jet of bleeding was identified from a lumbar artery.

**Figure 4:**
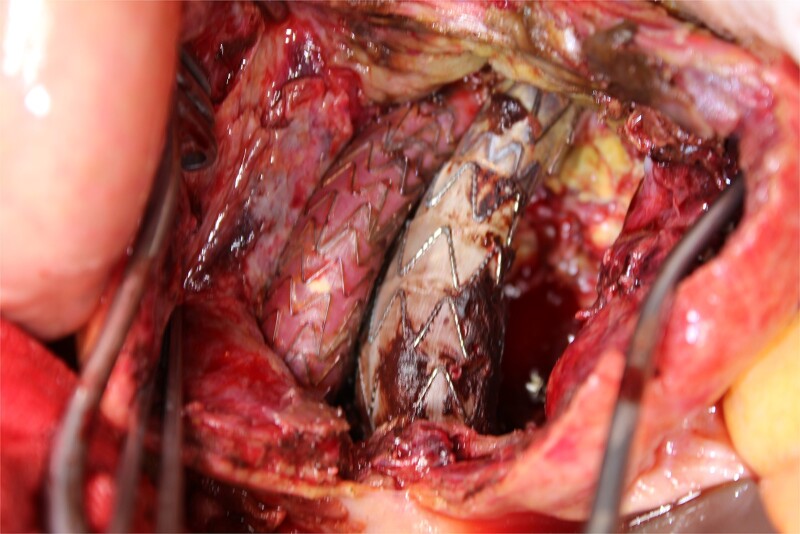
A photo taken from the caudal side of the patient. No endoleaks other than the type II endoleak were observed.

**Figure 5:**
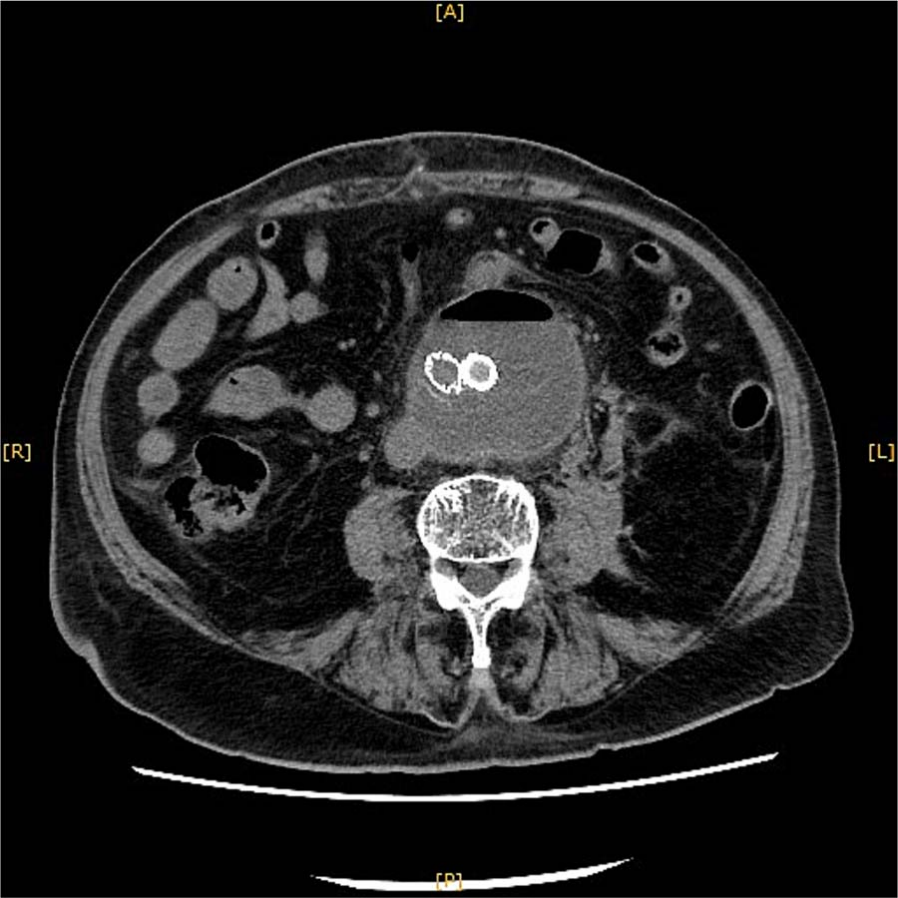
A plain CT on POD 14 showed a reduction in the aneurysm size with no apparent leaks.

This time, he was brought to the emergency department with a chief complaint of abdominal pain. His medical history includes Stage 4–5 chronic kidney disease (CKD), with an estimated glomerular filtration rate (eGFR) of approximately 15 mL/min/1.73 m^2^, secondary to nephrosclerosis and diabetic nephropathy, as well as persistent atrial fibrillation, which was treated with warfarin. Upon admission, laboratory results revealed a hemoglobin level of 6.2 g/dl and a prothrombin time—international normalized ratio (PT-INR) of 6.05. Contrast-enhanced CT demonstrated an AAA measuring 118 × 107 mm, enlarged from 98 × 94 mm 2 months prior, and left retroperitoneal hematoma. IMA and one pair of lumbar arteries were enhanced only in the delayed phase, but blood flow into the aneurysm was not clearly observed (Fig. 1). Given that the landing zones and overlaps of the stent grafts were satisfactory (Fig. 2) and that no type I or type III endoleaks were identified, a ruptured AAA due to TIIEL from the lumbar artery or IMA was highly suspected. We administered 3000 IU/kg of lyophilized human prothrombin complex concentrate in response to the significant prolongation of PT-INR and proceeded with open surgery for ligation of the feeding artery and aneurysmorrhaphy. Intraoperatively, no pulsation was found on the aneurysm. Incision of the aneurysm revealed a large amount of thrombus, which was removed, and then pulsatile bleeding was identified from a lumbar artery (Fig. 3), the ostium of which was sutured from within the aneurysm. This was consistent with the left lumbar artery of the pair enhanced on the preoperative CT and was suspected to be responsible for the rupture. Oozing was also noted from several other lumbar arteries, which were similarly sutured closed. No leak was observed from the proximal or distal ends, nor from the junctions of the stent grafts, and no damage to the stent grafts was observed (Fig. 4). Following this, aneurysmorrhaphy was performed, and the abdomen was closed. The postoperative course was uneventful. A plain CT on postoperative day (POD) 14 showed a reduction in the aneurysm size with no apparent leaks (Fig. 5). The patient was discharged home on POD 24. Although the preoperative eGFR was 8.8 ml/min/1.73 m^2^, no postoperative deterioration was observed. It gradually improved back to baseline, and dialysis was not required during the follow-up period.

## Discussion

TIIEL resolves spontaneously within 6–12 months in ~80% of cases [6, 7]. However, it is associated with aneurysm sac enlargement in 17.1% of cases, and some may subsequently develop type I endoleak [6]. A systematic review found that while 0.9% of all TIIELs ruptured, over one-third occurred without concurrent aneurysm expansion [5]. Accordingly, intervention for TIIEL should be considered only in symptomatic cases or when aneurysm expansion exceeds 10 mm [8]. In this case, the initial EVAR was performed 11 years ago at another hospital for an unruptured AAA measuring 54 × 52 mm. Five years later, type Ib endoleak and aneurysmal expansion to 81 × 82 mm were noted, leading to additional endovascular treatment. Since then, due to the patient’s relocation, close follow-up had been discontinued. A CT scan 2 months before the rupture revealed further enlargement to 98 × 94 mm, with no apparent type I or type III endoleaks. Given the stage 4–5 CKD, additional imaging with contrast media was not performed. Generally, anticoagulant therapy is recognized as a risk factor for aneurysm enlargement due to TIIEL [9]. In our case, excessive prolongation of PT-INR as a result of warfarin therapy may have contributed to the rupture. Treatment options for TIIEL include transcatheter or translumbar embolization, percutaneous ultrasound-guided thrombin injection, conversion to open repair, and laparoscopic clipping [10]. In this scenario, an open surgical approach was selected since further deterioration of kidney function could possibly occur with contrast-based intervention. Regarding the prevention of TIIEL itself, an RCT reported the efficacy of pre-emptive embolization for patients at risk of TIIEL (patent IMA ≥ 3 mm, lumbar arteries ≥2 mm) [11]. In this case, the lumbar artery suspected to be responsible for the rupture had a diameter of 2.08 mm on the CT before the initial EVAR, but no specific intervention was performed. Although there is no strong evidence for when intervention is indicated for TIIEL, this case suggests that early intervention should be considered even in the absence of other types of endoleaks, especially under circumstances similar to those presented here.
